# Spontaneous Retroperitoneal Haemorrhage: Efficacy of Conservative Management and Embolisation

**DOI:** 10.1007/s00270-023-03359-4

**Published:** 2023-01-31

**Authors:** Matthew Lukies, Jacob Gipson, Sia Yang Tan, Warren Clements

**Affiliations:** 1 Department of Radiology, Alfred Health, 55 Commercial Road, Melbourne, VIC 3004 Australia; 2 Department of Diagnostic and Interventional Imaging, KK Women’s and Children’s Hospital, Singapore, Singapore; 3 Department of Surgery, Monash University, Melbourne, VIC Australia; 4 National Trauma Research Institute, Melbourne, VIC Australia

**Keywords:** Retroperitoneal haemorrhage, Spontaneous retroperitoneal haemorrhage, Embolisation

## Abstract

**Purpose:**

To assess the efficacy of conservative management and embolisation in patients with spontaneous retroperitoneal haemorrhage.

**Methods:**

Single-centre retrospective case–control study of patients with spontaneous retroperitoneal haemorrhage treated conservatively or with embolisation. Patients aged ≥ 18 years were identified from CT imaging reports stating a diagnosis of retroperitoneal haemorrhage or similar and images reviewed for confirmation. Exclusion criteria included recent trauma, surgery, retroperitoneal vascular line insertion, or other non-spontaneous aetiology.

Datapoints analysed included treatment approach (conservative or embolisation), technical success, clinical success, and mortality outcome.

**Results:**

A total of 54 patients met inclusion criteria, who were predominantly anticoagulated (74%), male (72%), older adults (mean age 69 years), with active haemorrhage on CT (52%). Overall mortality was 15%. Clinical success was more likely with conservative management (36/38) than embolisation (9/16; *p* < 0.01), and all-cause (1/38 vs 7/16; *p* < 0.01) and uncontrolled primary bleeding (1/38 vs 5/16; *p* < 0.01) mortality were higher with embolisation. However, embolised patients more commonly had active bleeding on CT (15/38 vs 13/16; *p* < 0.01), shock (5/38 vs 6/16; *p* < 0.04), and higher blood transfusion volumes (mean 2.2 vs 5.9 units; *p* < 0.01). After one-to-one propensity score matching, differences in clinical success (*p* = 0.04) and all-cause mortality (*p* = 0.01) remained; however, difference in uncontrolled primary bleeding mortality did not (*p* = 0.07).

**Conclusion:**

Conservative management of SRH is likely to be effective in most patients, even in those who are anticoagulated and haemodynamically unstable, with variable success seen after embolisation in a more unstable patient group, supporting the notion that resuscitation and optimisation of coagulation are the most vital components of treatment.

**Supplementary Information:**

The online version contains supplementary material available at 10.1007/s00270-023-03359-4.

## Introduction

Spontaneous retroperitoneal haemorrhage (SRH) is defined as retroperitoneal bleeding that occurs in the absence of an incident cause such as trauma, surgery, or vascular line insertion [1–4]. Thought to principally occur as a rare phenomenon in elderly anticoagulated patients, SRH has a low published incidence of under 0.6% but a relatively high overall mortality of up to 22% based on case series published to date [1, 3–6]. Contributing to the high mortality rate may be the commonly delayed diagnosis due to heterogeneous presenting clinical features, such as abdominal pain, hip pain, flank/back pain, haemodynamic decline, and lumbar plexus neuropathy, as well as reliance on imaging for definitive diagnosis. The classically elderly patient cohort also typically have multiple medical comorbidities [1–3, 6–9].

Conservative (medical) measures including fluid resuscitation, blood transfusion, and cessation or reversal of anticoagulation are the mainstay of management and are highly successful in haemodynamically stable patients with SRH, as demonstrated by previous retrospective comparative studies between conservative and embolisation management approaches [1, 5–7]; however, the optimal management approach in patients with haemodynamic instability is less clear. Patients with haemodynamic instability due to SRH have historically been treated with surgical exploration; however, this has become less common over recent decades with the uptake of embolisation as a minimally invasive endovascular treatment option [1–3, 5–7, 10–12]. Embolisation typically involves deployment of coils, gelatin sponge, or liquid agents such as N-butyl-cyanoacrylate (NBCA) into the offending arterial branch, commonly a lumbar or iliolumbar artery [3, 5, 11, 12].

The aim of this study was to compare the success rates of conversative management and embolisation in the treatment of SRH, including in those with haemodynamic instability.

## Methods

### Ethical Approval

Approval for this single-centre retrospective case–control study was granted by our institutional review board (number 379/22). No funding was provided for this study.

### Patient Selection

The study covered a 10-year period from 1 January 2012 to 1 January 2022. The Radiology Information System (RIS) was searched by the four members of the research team (two consultant radiologists, one radiology trainee and one resident doctor) to identify cases applying the following inclusion criteria:

- Age ≥ 18 years
- CT report text containing the term(s): ‘retroperitoneal haemorrhage’, ‘retroperitoneal haematoma’, ‘retroperitoneal bleed’, ‘retroperitoneal bleeding’, ‘psoas haemorrhage’, ‘psoas haematoma’, ‘psoas bleeding’, ‘iliopsoas haemorrhage’, ‘iliopsoas haematoma’, and/or ‘iliopsoas bleeding’
- CT images were reviewed to confirm the diagnosis of retroperitoneal haemorrhage
- CT images were reviewed to document the presence of active bleeding (blush of contrast) as a datapoint, including both cases with and without active bleeding at the time of CT

The electronic medical records were reviewed in conjunction with the imaging data, and the following exclusion criteria applied:

- Documented recent history (< 4 weeks) of trauma, surgery, retroperitoneal vascular line insertion, and/or coronary angiography
- Evidence or signs of recent trauma, surgery, retroperitoneal vascular line insertion, and/or coronary angiography on CT and/or other imaging
- Presence of any other clearly non-spontaneous aetiology

### Outcome Measures and Statistical Analyses

**Fig. 1:**
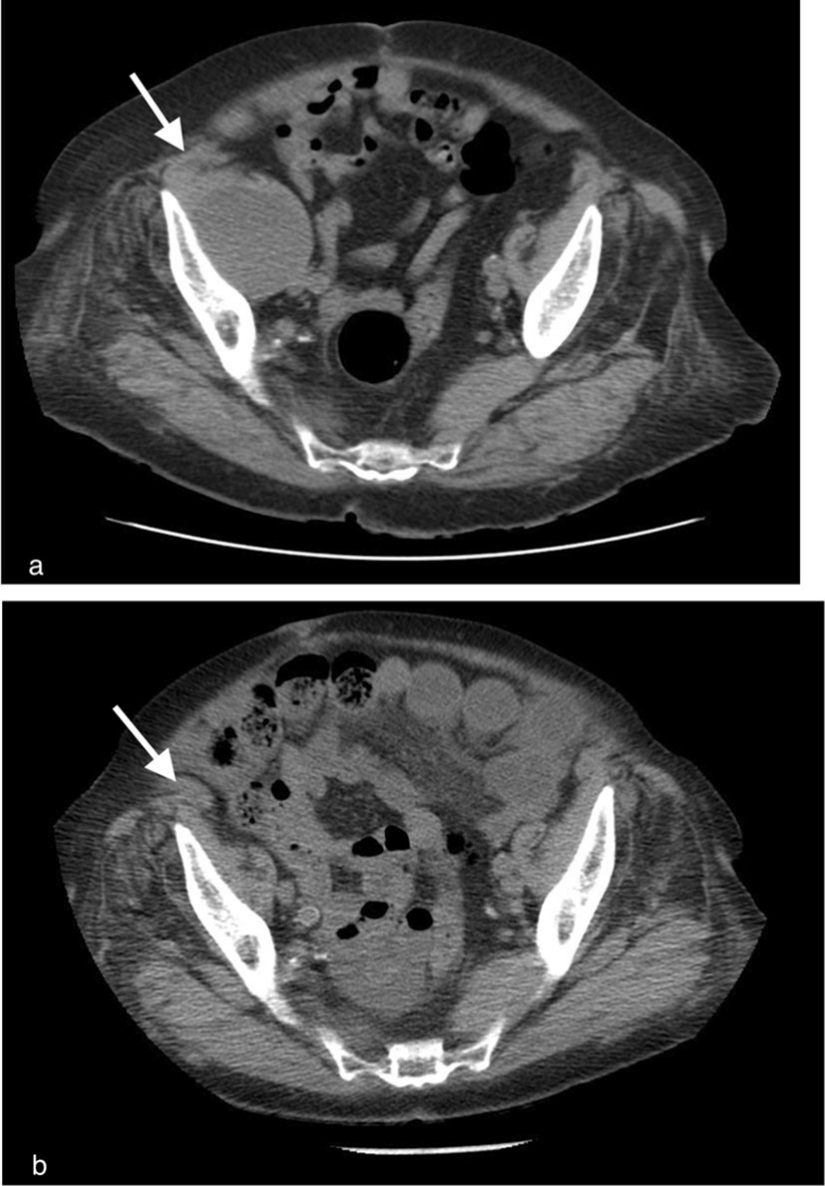
Spontaneous right iliacus retroperitoneal haematoma in a patient on warfarin (a; white arrow) managed conservatively with CT one year later showing complete resolution (b; white arrow)

**Fig. 2:**
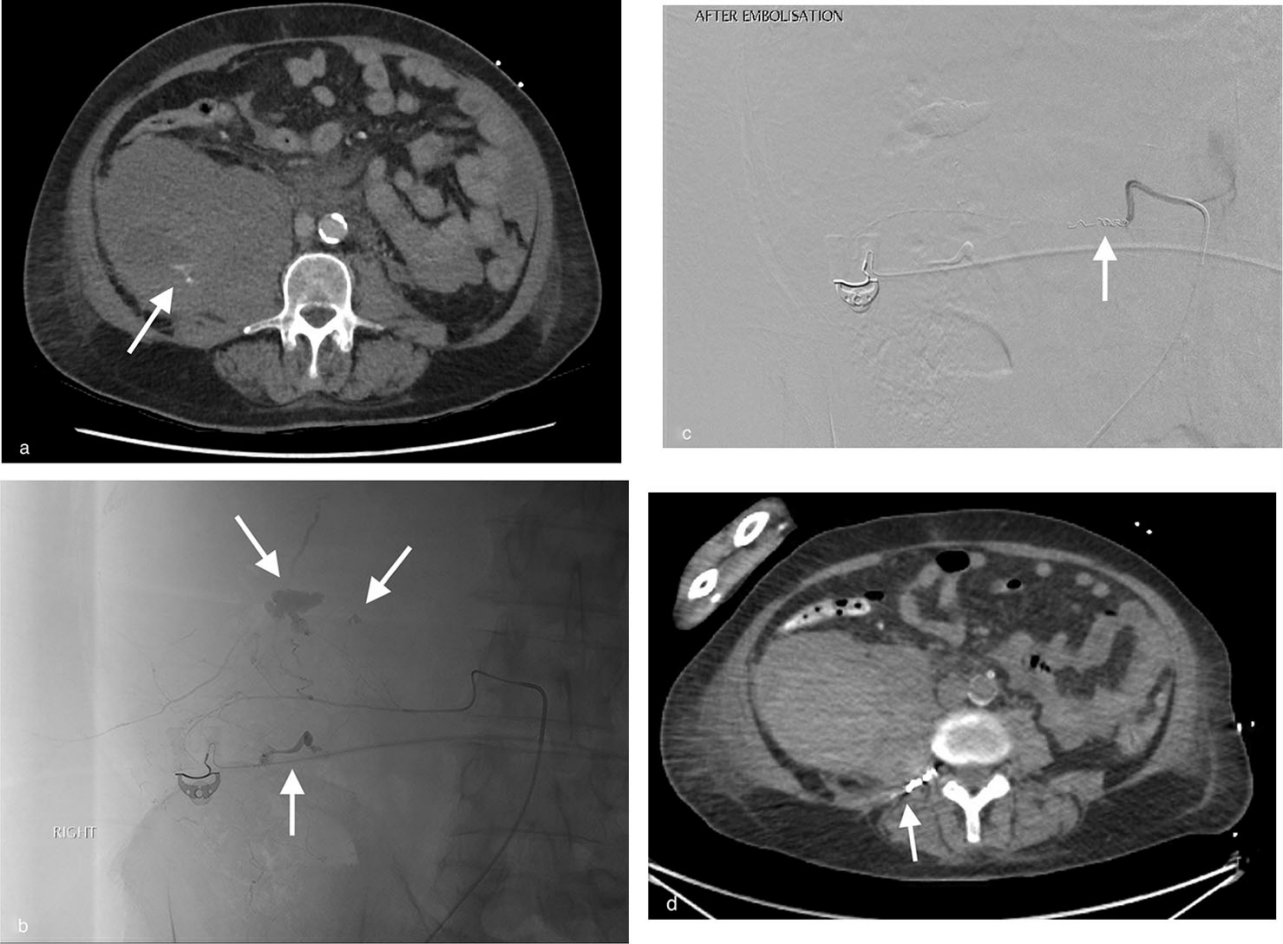
CT of the abdomen and pelvis in delayed contrast phase in a patient on therapeutic heparin showing a large right retroperitoneal haematoma with focus of contrast pooling/blush at the L3 level (**a**; white arrow); right L3 lumbar artery angiography with contrast injection via a 2.6-Fr microcatheter through a 5-Fr C2 parent catheter showing three foci of active extravasation (**b**; white arrows); angiography post embolisation with gelatin sponge and 2-mm coils showing stasis of flow (**c**; white arrow); CT of the abdomen without contrast three days later showing the coils in situ and stable haematoma size (**d**; white arrow)

From the electronic medical records, the following datapoints were extracted and recorded based on values at time of presentation with SRH: age, sex, blood pressure, heart rate, haemoglobin (Hb) level, platelet level, INR, medications (anticoagulation, antiplatelet, antihypertensive), COVID-19 status (2020 onwards), blood transfusion volume (within first 24 h), primary treatment method (conservative or embolisation), technical and clinical success rates, and outcome (survival, mortality). Primary treatment method referred to the treatment approach implemented within the first 24 h; specifically, conservative management implies that fluid resuscitation, blood transfusion, and cessation or reversal of anticoagulation were implemented, without embolisation or surgery within the first 24 h (Fig. 1), whereas embolisation implies that endovascular intervention was performed within 24 h of diagnosis in addition to conservative measures (Fig. 2). Technical success at embolisation was defined as cessation of flow through the offending vessel(s) on digital subtraction angiography. Clinical success was defined as absence of clinically significant rebleeding within 2 weeks of treatment. Technical and clinical failures were defined as failure to achieve cessation of flow through the offending vessel and clinically significant rebleed within 2 weeks, respectively. Data were summarised in Microsoft Excel and analysed using R software for tests of significance (t-test, Chi-square test, and Mann–Whitney U test). One-to-one propensity score matching was performed based on propensity scores from baseline characteristics (acute shock, active bleed on CT, and blood transfusion units) in a multivariable logistic regression model.

### Embolisation Protocol

Embolisation procedures were performed by locally credentialled specialist interventional radiologists and/or interventional radiology fellows (trainees) under direct supervision (total pool of 9 different operators), with experience ranging from 1 to 18 years. Procedural technique typically involved arterial access via the right common femoral artery, insertion of a 5- or 6-Fr vascular sheath, aortic digital subtraction angiography via a pigtail catheter, then selective angiography via a 4- or 5-Fr diagnostic catheter and a 2.7-Fr microcatheter to achieve more distal access. The embolic agents deployed were gelatin sponge, coils, and NBCA glue, selected at the discretion of the operator.

## Results

**Fig. 3:**
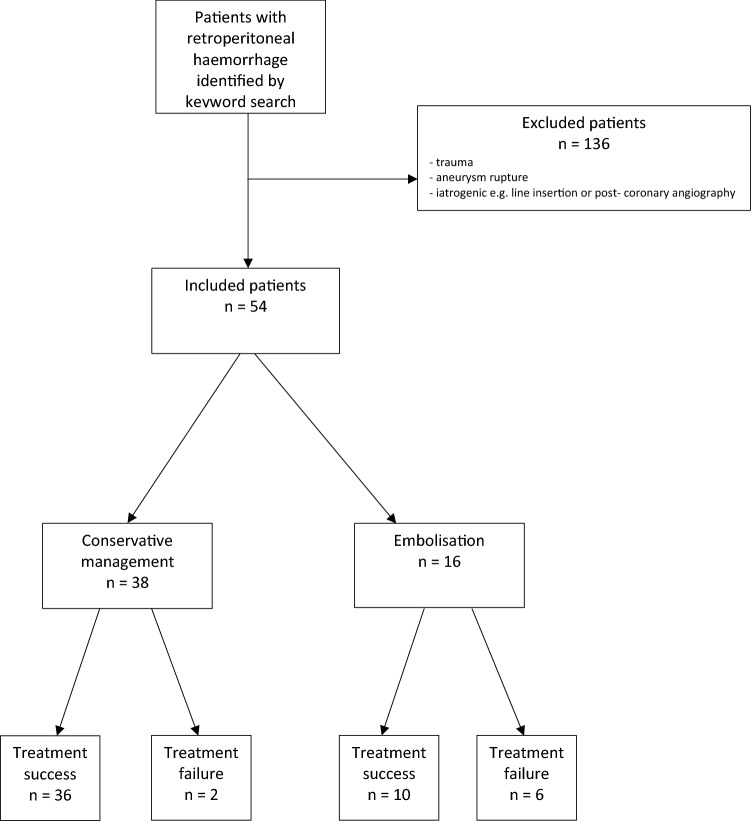
Flow chart showing patient recruitment into the study

A total of 190 patients were identified by the initial RIS search; after exclusion, a final cohort of 54 patients met inclusion criteria as shown in Fig. 3. The 54 included patients were 72% (*n* = 39) male and of mean age 69 years (standard deviation (SD) 12 years). Seventy per cent (*n* = 38) of patients were taking anticoagulant medication, most commonly warfarin (33%; *n* = 18). Fifty-six per cent (*n* = 30) were taking antihypertensives, with 35% (*n* = 19) taking two or more agents. Two patients had COVID-19 at the time of SRH. Twenty per cent (*n* = 11) had acute shock (as defined by shock index > 1; where shock index = heart rate divided by systolic blood pressure), 37% (*n* = 20) had haemoglobin level < 8 g/dL, and 30% (*n* = 16) had INR ≥ 2, and 27% (*n* = 15) had significant renal impairment (eGFR < 30 mL/min/1.73 m^2^). CT showed active haemorrhage (as defined by blush of contrast) in 52% (*n* = 28) of cases. Twenty-six patients (48%) received more than 3 units of PRBCs during the first 24 h of treatment, with 4 (7.4%) receiving 10 or more units.

Of the 38 patients in whom primary management was conservative, clinical success was seen in 36 (94.7%) (Table 1). One patient underwent subsequent technically and clinically successful embolisation for rebleed after initial conservative management, and survived, and another patient died after a decision to palliate. Embolisation was the primary management in 16 patients, with all 16 cases technically successful. Embolic agents used were gelatin sponge (7; 44%), coils (9; 56%), and glue (1; 6%), including cases utilising a combination of the above agents. Embolisation was clinically successful in 9 patients (56.3%). Four patients had ongoing instability despite embolisation and died. Two patients proceeded to surgical haemostasis for ongoing bleeding after embolisation, which was successful in one and unsuccessful (death) in the other, and one had initial clinical success but delayed rebleeding resulting in death. One patient died more than 2 weeks after embolisation from overwhelming bacteraemia, without further bleeding.

**Table 1:** Overall group comparison between conservative management and embolisation

	Conservative management	Embolisation	*p* value
Patients	38	16	N/A
Age			
Mean (range), years	69 (42–98)	71 (43–86)	0.46
Sex			
Male	26 (68%)	13 (81%)	0.33
Female	12 (32%)	3 (19%)	
Anticoagulation therapy *Agents:* Prophylactic enoxaparin Therapeutic enoxaparin Direct oral anticoagulant (DOAC) warfarin Heparin (unfractionated or infusion) More than one of the above agents	27 (71%) 2 5 1 14 7 3	11 (69%) 0 5 1 4 2 2	0.86
Antiplatelet therapy (aspirin and/or clopidogrel)	18 (47%)	6 (38%)	0.51
Antihypertensive therapy Single agent More than one agent	20 (52%) 7 13	10 (63%) 4 6	0.51
eGFR < 30	10 (26%)	5 (31%)	0.71
Active bleed on CT	15 (39%)	13 (81%)	< 0.01
Shock Index* > 1	5 (13%)	6 (38%)	0.04
Haemoglobin (g/dL); mean (SD)	9.11 (2.47)	8.45 (1.53)	0.33
Platelets (plts/microlitre); mean (SD)	239 (109)	294 (113)	0.10
INR; mean (SD)	2.24 (1.8)	1.76 (0.84)	0.33
Blood transfusion units; mean (SD)	2.2 (2.1)	5.9 (4.4)	< 0.01
Treatment clinical success	36 (94.7%)	9 (56.3%)	< 0.01
Uncontrolled primary bleeding Mortality	1	5	< 0.01
Delayed rebleeding mortality (≥ 2 weeks)	0	1	N/A
All-cause mortality (within 30 days)	1	7	< 0.01

*eGFR* Estimated glomerular filtration rate (mL/min/1.73m^2^). *INR* International normalised ratio. Shock Index = heart rate (HR) divided by systolic blood pressure (SBP)

Patients treated with embolisation were significantly more likely to have had active bleeding on CT and acute shock (shock index > 1) and received a significantly higher volume of blood transfusion in the first 24 h of care (Table 1). Clinical success was significantly more likely in those who had conservative management compared to embolisation, and both all-cause mortality and mortality due to uncontrolled primary bleeding were more likely with embolisation. After one-to-one propensity score matching, treatment clinical success and all-cause mortality remained significantly different; however, the difference in mortality due to uncontrolled primary bleeding was no longer significant (Table 2). These significant differences remained when analysing only those with active bleeding on CT (Table 3).

**Table 2:** Group comparison between conservative management and embolisation after one-to-one propensity score matching

	Conservative management	Embolisation	*p* value
Patients	16	16	N/A
Age			
Mean (range), years	67 (56–78)	71 (43–68)	0.27
Sex			
Male	11 (69%)	13 (81%)	0.43
Female	5 (31%)	3 (19%)	
Anticoagulation therapy	14 (88%)	11 (69%)	0.20
Antiplatelet therapy	7 (44%)	6 (55%)	0.71
Antihypertensive therapy	9 (56%)	10 (63%)	0.26
eGFR < 30	3 (19%)	5 (31%)	0.41
Active bleed on CT	11 (69%)	13 (81%)	0.41
Shock Index* > 1	6 (38%)	6 (38%)	1
Haemoglobin (g/dL); mean (SD)	8.08 (1.97)	8.45 (1.53)	0.55
Platelets (plts/microlitre); mean (SD)	265 (131)	294 (113)	0.51
INR; mean (SD)	2.63 (2.14)	1.76 (0.84)	0.14
Blood transfusion units; mean (SD)	3.5 (2.4)	5.9 (4.4)	0.06
Treatment clinical success	14 (87.5%)	9 (56.3%)	0.04
Uncontrolled primary bleeding mortality	1	5	0.07
Delayed rebleeding mortality (≥ 2 weeks)	0	1	N/A
All-cause mortality (within 30 days)	1	7	0.01

*eGFR* Estimated glomerular filtration rate (mL/min/1.73m^2^). *INR* International normalised ratio. Shock Index = heart rate (HR) divided by systolic blood pressure (SBP)

**Table 3:** Subgroup comparison of patients with active bleeding on CT

	Conservative management	Embolisation	*p* value
Patients	15	13	N/A
Age			
Mean (range), years	62 (42–78)	69 (43–86)	0.14
Sex			
Male	10 (67%)	11 (85%)	0.27
Female	5 (33%)	2 (15%)	
Anticoagulation therapy	11 (73%)	8 (62%)	0.51
Antiplatelet therapy	7 (47%)	4 (31%)	0.39
Shock Index* > 1	3 (20%)	5 (38%)	0.42
Haemoglobin (g/dL); mean (SD)	8.68 (2.45)	8.78 (1.44)	0.90
Platelets (plts/microlitre); mean (SD)	248 (112)	296 (111)	0.28
INR; mean (SD)	2.1 (2.0)	1.6 (0.55)	0.42
Blood transfusion units; mean (SD)	2.9 (2.6)	6.5 (4.5)	0.02
Treatment clinical success	13 (87%)	7 (54%)	0.06
Uncontrolled primary bleeding mortality	1	5	0.04
Delayed rebleeding mortality (≥ 2 weeks)	0	1	N/A
All-cause mortality (within 30 days)	1	6	0.02

*INR* International normalised ratio. Shock Index = heart rate (HR) divided by systolic blood pressure (SBP)

Overall all-cause mortality in the entire patient cohort was 15% (8/54), with the most likely cause in 6 the uncontrolled primary bleeding (including one where a decision was made for non-intervention and palliation), delayed rebleeding in 1, and sepsis in 1. Of the deceased patients, all but 1 had likely contributing medical comorbidities including coronary artery disease, congestive cardiac failure, bleeding diatheses, and sepsis. Comparing fatal and non-fatal cases, fatal cases were more likely to have presented with a shock index > 1 (odds ratio: 2.9, *p* = 0.04) and received a significantly higher number of red blood cell transfusion units (*p* < 0.01) (Table 4). Additionally, all 8 fatal cases showed active bleeding on CT (odds ratio: 2.3) and 4 had bacteraemia at the time of death.

**Table 4:** Comparison of non-fatal versus fatal cases

	Non-fatal	Fatal	*p* value
Patients	46	8	N/A
Age			
Mean (range), years	69 (42–86)	69 (52–86)	0.86
Sex			
Male Female	32 (70%) 14 (30%)	7 (87%) 1 (13%)	0.30
Anticoagulation therapy	35 (76%)	6 (75%)	0.94
Antiplatelet therapy	21 (47%)	3 (38%)	0.67
Antihypertensive therapy	24 (52%)	6 (75%)	0.23
eGFR < 30	13 (28%)	2 (25%)	0.85
Active bleed on CT	20 (43%)	8 (100%)	N/A
Shock Index* > 1	8 (17%)	4 (50%)	0.04
Haemoglobin (g/dL); mean (SD)	8.97 (2.35)	8.56 (1.25)	0.63
Platelets (plts/microlitre); mean (SD)	250 (112)	289 (96.3)	0.37
INR; mean (SD)	2.1 (1.7)	1.7 (0.57)	0.47
Blood transfusion units; mean (SD)	2.7 (2.9)	6.5 (3.5)	< 0.01

*eGFR* Estimated glomerular filtration rate (mL/min/1.73m2). *INR* International normalised ratio. Shock Index = heart rate (HR) divided by systolic blood pressure (SBP)

## Discussion

This study supports the notion that most patients with SRH can be safely treated conservatively [1, 3, 5–7]. While most patients treated conservatively were haemodynamically stable, conservative treatment was also successful in the 13.2% of patients initially unstable, 100% of whom were taking therapeutic anticoagulation. This is comparable to results in the study by Warren et al. [1] where the majority of anticoagulated patients with SRH were successfully managed with transfusion and cessation/reversal of anticoagulation, including patients with shock. These findings suggest that embolisation in SRH may be best reserved for patients with ongoing bleeding despite adequate reversal of anticoagulation agents, or patients who were not anticoagulated to begin with and are haemodynamically unstable.

Groups in the present study were not congruent, with embolised patients more likely to have been unstable and/or received a high blood resuscitation volume, and were not matched even after propensity score matching, particularly regarding blood transfusion volume. The lower success rate in the embolisation group was likely contributed to by underlying coagulopathy, which has been shown to develop in the setting of major blood loss [13]. Extrapolating from trauma literature, the most effective way to treat and prevent coagulopathy in acute blood loss is large volume transfusion of packed red blood cells, fresh frozen plasma, and platelets [14]. However, large volume transfusion has been associated with multi-organ failure and infection [15], which may account for the high rate of bacteraemia and high mortality in this SRH group.

SRH has traditionally been thought to occur predominantly in elderly patients on anticoagulation medications, with an overall low incidence but high mortality. The mean age of 69 years in the present cohort is closely concordant with previous reports, as are the percentages of patients with therapeutic anticoagulation (70%), INR ≥ 2.0 (30%) significant renal impairment (eGFR < 30; 22%), and two or more antihypertensive medications at the time of SRH (35%), reflecting the typically elderly and medically comorbid cohort [1–5]. Of recent interest, COVID-19 infection was present in two non-fatal cases and has been suggested as a possible risk factor given the coagulopathy and vasculopathy of the novel coronavirus [16]. The overall mortality of 15% in the present cohort is comparable to the range of 12% to 22% in recent published series with similar rates of embolisation [1, 3, 5, 6, 10].

There are limitations of this study to acknowledge. Firstly, the sample size is relatively small for the period of time assessed, but similar to other published series given the low incidence of SRH [5, 11, 12]. Secondly, the retrospective design relying on historical records limits the reliability of inferences made about risk factors and treatment outcomes in this cohort. Thirdly, as patients with SRH are typically elderly with a large number of significant medical comorbidities, it is often difficult to precisely determine cause of death, which may explain why there is variation in mortality attributable to the SRH in recent published literature but comparable overall mortality rates.

## Conclusion

This study showed that conservative management of SRH may be effective in most patients, even in those who are haemodynamically unstable but therapeutic anticoagulated. It supports the notion that resuscitation and optimisation of coagulation are the most vital components of treatment in SRH. Embolisation had varying success in our cohort, with significantly higher mortality; however, this was likely because patients who underwent embolisation were more commonly those with acute shock, high transfusion volumes, and active haemorrhage on CT. As shown in other studies, mortality in SRH is high, and goals of treatment should include addressing the coagulopathy and not just arrest of haemorrhage.

## Supplementary Information

Below is the link to the electronic supplementary material.

Supplementary file1 (DOC 99 kb)
